# Ethical and Quality of Care–Related Challenges of Digital Health Twins in Care Settings for Older Adults: Scoping Review

**DOI:** 10.2196/73925

**Published:** 2025-10-28

**Authors:** Md Shafiqur Rahman Jabin, Ayesha Mirza, Adaobi Ilodibe, Tillal Eldabi, Emilia Vann Yaroson

**Affiliations:** 1 Department of Medicine and Optometry, Linnaeus University, Universitetsplatsen 1, Kalmar, 392 34, Sweden, 44 7915 673 612; 2 Faculty of Health Studies, University of Bradford, Bradford, United Kingdom; 3 Faculty of Engineering and Digital Technologies, University of Bradford, Bradford, United Kingdom; 4 School of Management, University of Bradford, Bradford, United Kingdom; 5 Management School, Sheffield University, Sheffield, United Kingdom

**Keywords:** patient safety, health equity, effectiveness, equality, accessibility, social care, data security, right to privacy, patient consent, overdiagnosis, PRISMA, Preferred Reporting Items for Systematic Reviews and Meta-Analyses

## Abstract

**Background:**

Digital health twins (DHTs) have been evolving with their diverse applications in medicine, particularly in care settings for older adults, in response to the increasing demands of older adults. Despite its numerous benefits, the optimal implementation of DHTs has faced several challenges, particularly in terms of ethics and quality of care. Given the continuous rise in the need for such care and the evident potential for DHTs to meet these needs, this review seeks to identify and address the gaps in research knowledge to enhance DHT implementation.

**Objective:**

The review aims to compile and synthesize the best available evidence regarding the issues associated with quality of care, the ethical implications of DHTs, and the strategies undertaken to overcome those challenges in care settings for older adults.

**Methods:**

The review followed the Joanna Briggs Institute (JBI) methodology as a guide. The published studies were searched through CINAHL, MEDLINE, JBI, and Web of Science. The unpublished studies were searched through Mednar, Trove, OCLC WorldCat, and Dissertations and Theses. Studies published in English from 2016 were considered. This review included studies of older individuals (aged 60 years and older) undergoing care delivery associated with DHTs and respective care providers. The concept involved the application of technology, and the context included studies based on care settings for older adults. A broad scope of evidence, including quantitative, qualitative, text, and opinion studies, was considered. In addition, 2 independent reviewers screened the titles and abstracts and reviewed the full text.

**Results:**

The results will be presented in a PRISMA (Preferred Reporting Items for Systematic Reviews and Meta-Analyses) flow diagram. A total of 2 draft charting tables were developed and presented. A summary of the characteristics of the included studies was then described in terms of location, study sites, timing, participants, and outcomes measured or phenomena of interest. A result-based convergent (integrated) synthesis design was used to identify 5 key challenges. Those challenges included (1) data security and privacy concerns, (2) equity and accessibility of health care, (3) effectiveness concerning context, timing, and location, (4) ethical implications regarding autonomy, consent, and overdiagnosis, and (5) the impact of DHTs on health care workflows and provider workload.

**Conclusions:**

The studies reviewed reveal several critical characteristics regarding the implementation of DHT technologies and their associated ethical considerations, particularly in terms of safety, equity, timing, location, participant characteristics, and workflow impact. The implications of these challenges emphasize the necessity for more practical ethical guidelines and policy frameworks to mitigate the potential risks associated with DHT application in older care. Further research should be conducted to examine other dimensions of the quality of care, such as access, timeliness, acceptability, and appropriateness.

## Introduction

Digital health, with its tools for telemedicine, remote monitoring, medication management, and social connection, is a reassuring presence in the care of older adults. It is not just about enhancing their health, independence, and quality of life, but also about promoting healthy aging. This is achieved through the promotion of preventive care, disease self-management, and the personalized interventions that address the specific needs of older adults [1,2]. Digital health interventions can be tailored to individual needs and abilities (personalized care), addressing specific health concerns like falls, sarcopenia, and mental health challenges to support independence and well-being [3,4].

The advent of digital health twins (DHTs) marks a pivotal evolution in health care and social care, particularly in the management of care for older adults. These sophisticated virtual representations simulate individual health profiles by integrating extensive datasets, promising personalized health and social care interventions that can significantly enhance patient outcomes [5,6]. Despite the considerable advantages that DHTs present—improvements in effectiveness, safety, and accessibility of care—their implementation raises critical ethical dilemmas and challenges related to the quality of care that necessitate rigorous examination [7,8]. DHTs can be more than just a digital replica or a virtual model of patients (physical twins); they can be a sophisticated representation designed to faithfully mirror the real-world system in real time, analyze their behavior, and provide predictive insights using advanced simulation, machine learning, and reasoning to inform decision-making. The analytical and predictive capability of a digital twin makes it distinct from a dummy replica of the physical system [9,10]. DHTs are generated from multimodal patient data, population data, and real-time updates on patient and environmental variables [11]. For the convenience of the reader, the term “digital health twins” (DHTs) has been consistently maintained throughout the article. However, several other related terms, such as “digital twins” and “personal digital twins,” have also been used to depict the findings, thereby maintaining the originality and credibility of the included studies, primarily in the results section and tables.

DHTs are considered the pinnacle of personalized health care due to their ability to create a virtual replica of a patient’s health information, created using various data sources—from genetic information to real-time biometric data [6,12]. One of the primary concerns associated with DHTs is their potential to redefine established health norms. Creating personalized health models could inadvertently establish new benchmarks affecting the safety and effectiveness of health and social care interventions across diverse patient populations [13]. This technology has the potential to vastly improve the accuracy and efficiency of health care delivery, leading to better outcomes for patients and providers alike [12].

However, deploying such innovations must be approached with caution to mitigate risks related to data privacy and security, particularly among older adults who are often more susceptible to data breaches [14]. DHTs present heightened privacy and security risks compared to other technologies due to their reliance on sensitive, real-time patient data and their complex, interconnected nature. The continuous and urgent influx of personal health information, combined with the potential for remote access and manipulation, makes DHTs a prime target for cyberattacks and privacy breaches, highlighting the need for collaborative efforts among professionals to address this issue [15]. As digital health solutions become increasingly pervasive, the ethical ramifications surrounding data ownership, consent, and potential misuse warrant careful consideration [16,17].

DHTs can revolutionize personalized care planning. Numerous studies have demonstrated that these models can enhance the customization of interventions, leading to improved health outcomes [16]. However, the inherent personalization also introduces ethical considerations regarding data representation and equitable access to care. Bahrami et al [18] emphasized concerns regarding equitable access to tailored drug therapies, which may inadvertently exacerbate existing disparities in health and social care.

The potential of DHTs to facilitate early detection of health issues, such as dementia, is widely acknowledged [19]. These systems can enhance care safety and effectiveness through timely interventions and provoke significant privacy and autonomy concerns [20]. The ethical implications of using technology for health monitoring necessitate the development of robust frameworks that prioritize patient autonomy while promoting engagement with digital health solutions [21]. This highlights the importance of considering ethical aspects of DHT in health and social care.

Furthermore, the intersection of DHTs with traditional health and social care practices raises profound ethical questions concerning the equilibrium between potential benefits and harms. For instance, Lin et al [22] scrutinized the risk of overdiagnosis in colorectal cancer screening programs, highlighting the likely exacerbation of this issue by DHTs. The unintended consequences of digital health interventions include patient anxiety and unnecessary medical procedures, underscoring the necessity for thorough ethical scrutiny [23]. As health and social care transitions toward a data-driven paradigm, integrating emerging technologies must be guided by ethical principles that prioritize patient welfare.

Incorporating innovative technologies, including virtual reality (VR), within DHT frameworks opens new avenues for training and treatment [24]. While VR simulations can enhance health and social care training and improve treatment outcomes, they also present ethical challenges regarding informed consent and data use. Ensuring these technologies are used ethically and responsibly is paramount to maintaining public trust in digital health innovations.

This research investigates the ethical and quality of care-related challenges associated with DHTs in older adult care settings. A scoping review has been chosen because it is designed to map key concepts and examine the literature in a research area, providing an overview of the current evidence available. By synthesizing existing literature and identifying knowledge gaps, particularly as articulated in the protocol by Jabin et al [8], this study aims to provide a comprehensive understanding of the implications of DHTs. In doing so, it will underscore the necessity for frameworks that ensure technological advancements in health and social care do not compromise the fundamental ethical principles of equity, autonomy, and patient-centered care. This reinforces the need for ethical guidelines in the digital health field, where integrating novel technologies must be closely aligned with ethical considerations without compromising the quality of health and social care. Specifically, the review questions are:

- What problems are faced by older individuals (their family and relatives), and health and social care providers, associated with the application of DHTs in care settings for older adults?
- What are the documented issues related to the quality of care for older adults, such as safety, equity, effectiveness, and accessibility, concerning DHTs?
- What are the ethical challenges concerning the application of DHTs used in care settings for older adults?
- What strategies have been evaluated and implemented in care settings for older adults that address the challenges associated with DHTs?

## Methods

The proposed scoping review was conducted under the guidance provided by the Joanna Briggs Institute (JBI) methodology for scoping reviews [25]. The JBI methodology for scoping reviews offers a systematic and structured approach for mapping and reviewing vast areas of study.

### Eligibility Criteria

#### Overview

This scoping review included studies by following the population, concept, and context (PCC) mnemonics. These mnemonics were used as a guide (not a policy); therefore, the inclusion criteria of this scoping review included a detailed description of the types of participants, concepts, and context, as well as search strategies, data extraction, charting, analysis, and presentation of the results. The eligibility criteria are listed and described in detail (see Table 1 and Table S1 in Multimedia Appendix 1) of the original protocol published by Jabin et al [8].

**Table 1.** Quality of care, ethical challenges, and strategies of included studies.

Study reference (year)	Quality of care	Ethical challenges	Strategies
Bruynseels et al [12] (2018)	Redefines health norms, affecting safety and effectiveness	Inequality and discrimination risks	Governance for data transparency and privacy
Liu et al [26] (2019)	Enhances monitoring and prediction, affecting effectiveness and accessibility	—^a^	Cloud-based framework to improve care delivery
Chakshu et al [16] (2019)	Noninvasive health monitoring enhances safety and effectiveness	Not directly addressed	Semiactive digital twin model for health monitoring
Calderita et al [27] (2020)	CPS^b^ integration improves caregiving, potentially affecting safety and accessibility	Not directly addressed	CPS-AAL^c^ systems for quality-of-life enhancement
Vidal et al [28] (2020)	Care coordination through integrated living models (ILMs) and advancing noninvasive sensors for accurate health monitoring	Transparent and unbiased use of diverse health data and algorithms	Multidisciplinary collaboration, extending research across all age groups, and enhancing caregiver coordination through ILM
Barbiero et al [11] (2021)	Digital twin framework could improve personalized care plans	Personalization implicates ethical considerations	Framework as a strategy for preventative medicine
Jovanovic et al [29] (2021)	Affects health intervention effectiveness with vaccination strategies	Impact of information dissemination on ethics	Vaccination strategy simulation model
Kobayashi et al [19] (2021)	System for early dementia sign detection enhances care safety and effectiveness	Privacy and autonomy concerns	Early detection system for dementia care
Khan et al [30] (2022)	Unobtrusive sensors for data collection are relevant to safety and effectiveness	Privacy concerns due to data collection	Microwave sensing for nonintrusive data collection
Bahrami et al [7] (2022)	Tailored drug therapy digital twin addresses safety and effectiveness	Raises questions about equitable access	Physics-based digital twin for personalized medicine
Wickramasinghe et al [31] (2022)	Advocates for digital twins in dementia care for effectiveness and personalization	Not directly addressed	Clinical decision support model for dementia care
Sahal et al [32](2022)	PDTs (patient-delivered therapies) improve health care effectiveness.	Blockchain hints at addressing privacy	Reference framework for personalized health care
Zhou et al [33] (2022)	Metaverse in cognitive decline intervention could affect accessibility and effectiveness	Privacy and autonomy issues with meta-hospitals	Metaverse as a strategy for nonpharmacological interventions
Alves et al [5] (2022)	VR^d^ simulator for robot training impacts safety and effectiveness	Consent in data used for simulation training	VR simulation for training without patient data
Bahrami et al [18] (2023)	Effects of physiological features on pain relief, impacting effectiveness	Equity concerns with tailored therapy	Digital twin control of therapy for pain management
Bui [21] (2023)	Insights into elderly perceptions of DHT^e^ affect accessibility and acceptance	User acceptance implicates autonomy and privacy issues	Behavioral intention understanding for DHT adoption
Lin et al [22] (2023)	Assesses overdiagnosis in CRC^f^ screening, affecting safety and effectiveness	Raises ethical concerns about harm versus benefit	Digital twin approach to mitigate overdiagnosis
Zhao et al [20] (2023)	Assisted experience of seniors with technology affects accessibility and effectiveness	Ethical considerations of autonomy and equity	Digital twin remote collaboration enhancement
Thamotharan et al [23] (2023)	HDT^g^ for diabetes management impacts safety and effectiveness	Personalized treatment raises privacy and equity issues	HDT framework for individualized diabetes treatment
Cai et al [34] (2023)	STRIDE^h^ for ADRD^i^ risk evaluation improves effectiveness and safety in gait analysis	Privacy concerns with gait data collection	STRIDE with MDR^j^ and DL^k^ for noninvasive risk evaluation

a Not available.

b CPS: Cyber-Physical System.

c CPS-AAL: Cyber-Physical System for Ambient Assisted Living.

d VR: virtual reality.

e DHT: digital health twin.

f CRC: colorectal cancer.

g HDT: health digital twin.

h STRIDE: Specific context needed, often refers to "Strategies for Risk Identification and Development" or other frameworks.

i ADRD: Alzheimer disease and related dementias.

j MDR: multidrug resistance.

k DL: deep learning.

#### Participants

This review included studies of older individuals (aged 60 years and older) undergoing older care associated with DHTs, irrespective of gender and diversity, including age, ethnicity, socioeconomic status, disorders, and disability. Studies on caregivers (family or friends—paid or unpaid) and care providers (licensed or unlicensed) involved in older care concerning DHT were also included.

#### Concept

The key concept in this scoping review is the process and application of DHT. Studies evaluated the application of DHT involving older care providers, older individuals, or family, friends, and relatives.

#### Context

The scoping review considered studies in care settings for older adults associated with DHT, such as geriatric wards of primary health care, hospitals or clinics, old-age homes, nursing homes, care homes, and home care facilities for older individuals.

### Search Strategy

Databases were searched for both published and unpublished studies. The approach to searching for studies for a scoping review followed the standard 3-step method [8]. The first step involved an initial limited search of relevant databases, followed by an analysis of the text words in the title and abstract, as well as the index terms used to describe the article. The search for published studies included a 2-way search strategy. One way was to search the journal and reference databases, such as CINAHL, MEDLINE, JBI, and Web of Science. Another way was to search article-based (journal) databases, such as ACM Digital Library, IEEE Xplore, and BMJ Journals. The search for unpublished studies included Mednar, Trove, OCLC WorldCat, and Dissertations and Theses. A second search was undertaken across all included databases using all identified keywords and index terms. Additional search strategies, including citation searches for specific researchers or articles (eg, gold-standard articles) and chain searches (reviewing the reference lists of systematically selected articles), were included to complement the search for published and unpublished papers. Studies, such as reviews (systematic, scoping, and umbrella) and letters to editors, were excluded. Studies published in English were considered. While the precise origin of the specific phrase “digital health twin” is hard to pinpoint, the foundational concepts of digital twins began gaining significant traction in healthcare around 2016, driven by advancements in related fields like the internet of medical things and artificial intelligence [24].

### Types of Studies

This scoping review considered experimental and quasi-experimental study designs, including randomized controlled trials, nonrandomized controlled trials, before-and-after studies, and interrupted time-series studies. In addition, analytical observational studies, including prospective and retrospective cohort studies, case-control studies, and analytical cross-sectional studies, were also considered for inclusion. This review considered descriptive observational study designs, including case series, individual case reports, and descriptive cross-sectional studies for inclusion.

Qualitative studies were also considered, focusing on qualitative data, including, but not limited to, designs such as phenomenology, grounded theory, ethnography, qualitative description, and action research. Text and opinion papers regarding the benefits, challenges, and strategies to overcome the challenges posed by DHT were also considered, as the scoping review includes a broad scope of evidence.

### Source of Evidence Selection

Following the search, all identified citations were collated and uploaded into EndNote 20 (Clarivate Analytics), and duplicates were removed. Following a pilot test, titles and abstracts were then screened by 2 or more independent reviewers for assessment against the inclusion criteria for the review. Potentially relevant sources were retrieved in full, and their citation details were imported into a Microsoft Excel sheet instead of the JBI System for the Unified Management, Assessment, and Review of Information [35] due to a lack of resources. In addition, 2 independent reviewers assessed the full text of selected citations in detail against the inclusion criteria. Reasons for excluding sources of evidence in full text that did not meet the inclusion criteria were recorded and reported in the scoping review. Any disagreements that arose between the reviewers at each stage of the selection process were resolved through discussion or with the assistance of an additional reviewer (as an arbitrator). The search results and the study inclusion process were reported in full in the final scoping review and presented in the PRISMA-ScR (Preferred Reporting Items for Systematic Reviews and Meta-Analyses Extension for Scoping Reviews) flow diagram [36,37].

### Data Extraction

A total of 2 independent reviewers used a data extraction tool they had developed to extract data from the papers included in the scoping review (see Table 1). The data extracted included specific details about the participants, concept, context, study methods, and key findings relevant to the review questions.

A draft charting table was developed as a data extraction tool. While extracting data from each included evidence source, the charting table was modified and revised as necessary. The modifications were detailed in the scoping review. Any reviewer disagreements were resolved either by the reviewer or with the assistance of an additional reviewer (as an arbitrator).

We extracted variables to ensure alignment with our research questions, while some variables may emerge during full-text screening; therefore, it was essential to predefine the key data extraction elements (as the protocol is structured). Clearly outlining these variables ensured consistency in data collection, enhanced transparency in the scoping review methodology, and strengthened the study’s replicability. We also followed the JBI Manual for Evidence Synthesis as a guide, which provided a basic data extraction table template to ensure completeness, methodological consistency, and adherence to best practices in data extraction [38].

## Results

### Overview

The results are presented as a “map” of the data extracted from the included papers, in a tabular form (as necessary) and in a descriptive format that aligns with the review’s objective and scope. A clear explanation for each category is provided, accompanied by a narrative summary that describes how the results relate to the review’s objectives and questions. This scoping review synthesizes key findings from the literature on the application of DHTs in older adult care settings.

A result-based convergent synthesis design was used in which all types of studies, such as quantitative and qualitative, were analyzed and presented [39]. The findings were then integrated using convergent synthesis, which identified five key challenges. Those challenges included (1) data security and privacy concerns, (2) equity and accessibility of health care, (3) effectiveness concerning context, timing, and location, (4) ethical implications regarding autonomy, consent, and overdiagnosis, and (5) the impact of DHTs on health care workflows and provider workload.

### Search Strategies and Screening

**Figure 1.**
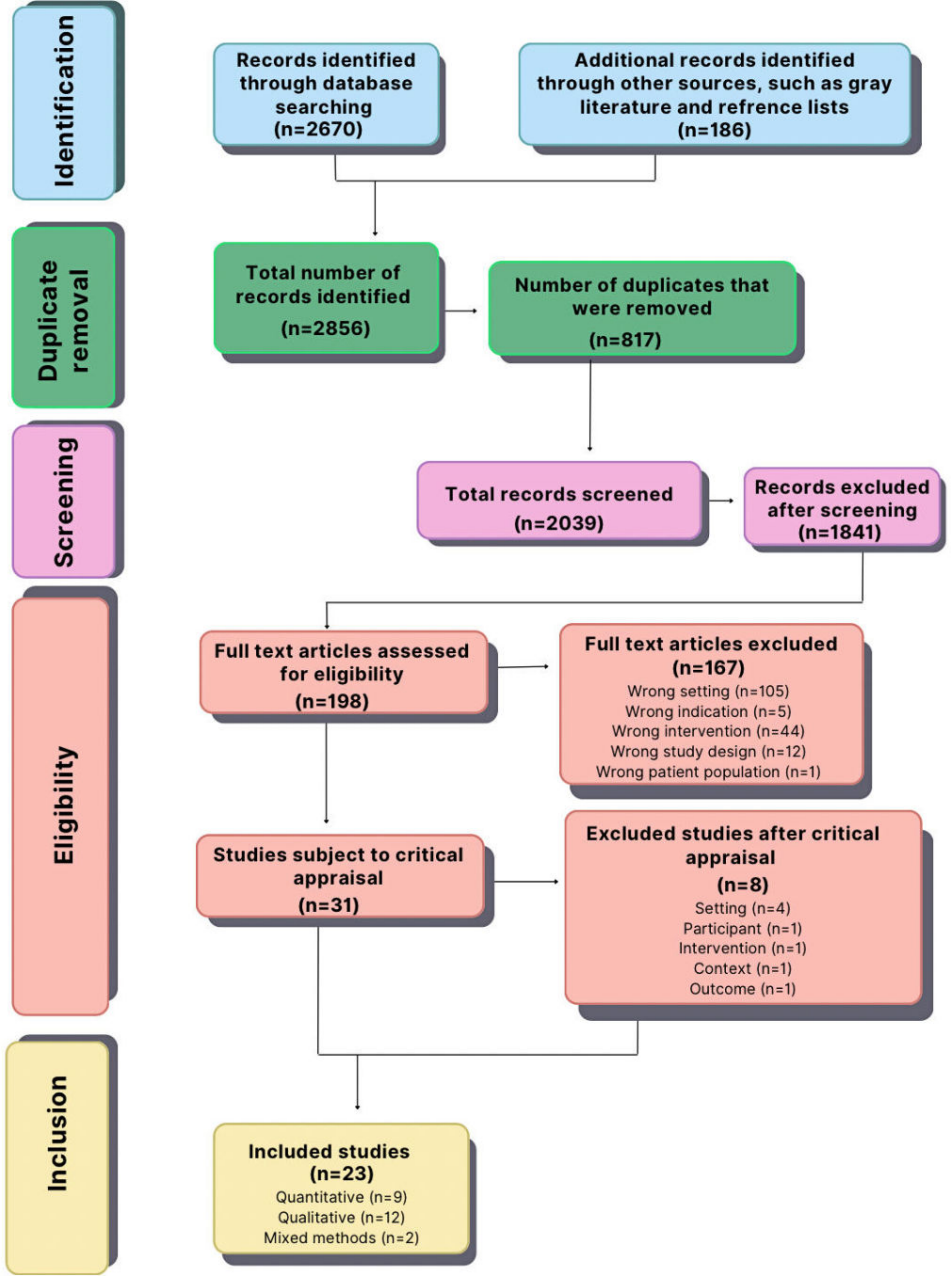
Preferred Reporting Items for Systematic Reviews and Meta-Analyses (PRISMA) flow diagram of the studies in the scoping review.

Figure 1 shows the results of the search strategy and inclusion of studies as a Preferred Reporting Items for Systematic Reviews and Meta-Analyses (PRISMA) flow diagram. The total number of studies identified was 856, which included searching bibliographic databases (n=786 studies) and searching for gray literature and other sources, such as reference lists of included studies (n=70 studies). After removing 344 duplicates, a total of 512 records were screened. The titles and abstracts of each of the 512 studies were screened independently by 2 reviewers (the principal author as the first reviewer of all studies and the contributing authors as second reviewers on a similar number of studies each); 354 studies were excluded after screening because they were not relevant to the review question. A full-text review assessing the eligibility of the remaining 158 studies resulted in 20 studies being advanced to the final list of included studies, while 138 studies were excluded (see Figure 1).

A search strategy using Boolean search strings in databases is provided in Table S2 in Multimedia Appendix 2, and the inclusion and exclusion criteria used during the review process are outlined in Table S3 in Multimedia Appendix 2.

### Summary of the Characteristics of the Included Studies

The studies reviewed emphasize the integration of DHTs in health care, offering a wide range of applications, including virtual simulations, disease monitoring, and health care delivery.

#### Location, Study Sites, and Timing

The studies vary in terms of location and timing (see Table S1 in Multimedia Appendix 1). For example, Bruynseels et al [12] explore the potential of DHTs in health care settings, but do not specify particular sites or study duration. On the other hand, Khan et al [30] use microwave sensors in a static care-home model, which likely involves specific care-home locations, showcasing a practical implementation of DHTs in elderly care. The studies span different periods, with some focusing on contemporary advancements [32] and others taking a longer-term view of digital health transformation [26]. All included studies encompass a range of countries (n=14) globally, with the majority originating from the United Kingdom and Europe. The publication of these 20 studies, spanning from 2018 to 2023, suggests that DHT has gained attention only recently.

#### Participants

Participant involvement varies across the studies (see Table S1 in Multimedia Appendix 1). For example, Jovanovic et al [29] focus on vaccination strategy simulations, meaning their study does not involve direct patient participation but relies on data for modeling purposes. In contrast, the model by Bahrami et al [7] drug diffusion in an online patient, suggesting that their study could involve patient-based data for the creation of DHTs. Studies like those delve into ethical issues related to using DHTs in care settings for older adults, which would likely involve patient consent and ethical considerations for vulnerable groups [12,22,28].

#### Outcomes Measured/Phenomenon of Interest

The outcomes measured in the studies often focus on the effectiveness of DHT technology in improving health care outcomes (see Table S1 in Multimedia Appendix 1). For example, Liu et al [26] assess the use of cloud-based DHTs for elderly health care services, measuring outcomes such as patient monitoring and health service optimization. In contrast, studies like the one by Cai et al [34] focus on the early detection of Alzheimer disease, aiming to measure how DHTs could assist in identifying disease risk early, ultimately improving diagnosis and treatment outcomes.

#### Claims and Findings

The reviewed literature consistently highlighted that DHTs and associated technologies offer transformative potential for improving health care delivery, particularly through personalization, predictive analytics, and real-time monitoring (see Multimedia Appendix 1). However, shared concerns about ethics, equity, and data privacy were repeatedly emphasized across the studies. For example, Bruynseels et al [12] argued that DHTs raise significant ethical, legal, and societal issues, particularly regarding equality and potential discrimination, which resonated with Chakshu et al [16], who, although focusing on noninvasive detection of carotid stenosis, implicitly align with Bruynseels and colleagues’ [12] ethical stance by advocating for procedural alignment and ethical validation in clinical application. Both stressed the need for patient autonomy and safeguarding rights in digital health innovation [12,16].

Similarly, the concerns around data privacy and security raised by Khan et al [30], who examined unobtrusive microwave sensors in care homes, were mirrored in Kobayashi et al [19], who discussed privacy and autonomy concerns in their dementia detection system. Both highlighted that the collection of personal health data—whether through sensors or DHTs—demands robust ethical and legal frameworks to protect vulnerable populations, particularly older adults [19,30].

In terms of personalized care and equity, Bahrami et al [7] proposed a physics-based digital twin for tailored drug therapy, focusing on personalized medicine, which aligned closely with Barbiero et al [11], who developed a general digital twin framework emphasizing modularity and scalability in representing biomedical data. Both studies demonstrated how, although helpful for precision treatment, personalized health care models could worsen disparities if access is not sufficiently addressed [7,11]. Wickramasinghe et al [31], who emphasized the necessity of inclusive clinical decision support in dementia care, share this issue.

Further illustrating a shared commitment to enhancing health care efficiency and safety, while acknowledging ethical concerns such as informed consent and data governance, is the integration of cutting-edge technologies, including VR simulators by Alves et al [5] and personal digital twins (PDTs) by Sahal et al [32]. Alves and colleagues’ [5] focus on online training environments and Sahal and colleagues’ [32] blockchain-enabled PDT framework were both operating in tandem, suggesting technological steps to ensure openness and confidence [5,32]. Sahal and colleagues’ [32] blockchain-enabled PDT framework and Alves’ emphasis on online training environments complemented each other by recommending technological measures to guarantee transparency and trust [5,32].

A notable similarity existed between Jovanovic et al [29] and Lin et al [22] in their use of simulation and modeling to guide public health interventions—Jovanovic et al through vaccination strategy simulations and Lin et al via overdiagnosis assessments in colorectal cancer screening [22,29]. Both emphasized the importance of data-driven decision-making to optimize health outcomes and minimize unintended consequences, such as overdiagnosis or inefficient resource allocation [22,29].

Finally, the user perception and adoption challenges explored by Bui [21], regarding older adults’ attitudes toward DHTs, were closely reflected in Zhao et al [20], who examined older users’ expectations of intelligent technologies. Both studies concluded that user acceptance was influenced by privacy concerns, ease of use, and perceived benefits, underlining the critical need for human-centered design in DHT solutions [20,21].

### Integrated Synthesis

This review identified a range of challenges based on the key characteristics of the included studies (see Table 1), which are briefly described hereinafter:

#### Safety Concerns and Data Privacy

Safety remains a primary concern in the deployment of DHTs, particularly regarding patient data security and privacy.

##### Risks to Patient Privacy

DHTs, as virtual representations of patients, relied on integrating extensive multimodal datasets [12], including health records, environmental data, and real-time monitoring information [5]. While these technologies offered the potential to improve safety through personalized health monitoring and predictive capabilities, they also raised significant risks to patient privacy.

##### Unauthorized Data Access and Misuse

Older adults are often more vulnerable to data breaches due to cognitive impairments or a lack of digital literacy [21], and require additional safeguards [34]. Studies by Liu et al [26] and Khan et al emphasized the potential for unauthorized data access and misuse [26], which could undermine trust in DHT technologies. This concern is amplified in older populations [30] who may lack familiarity with digital systems and are more susceptible to exploitation.

Protecting patient data through secure and transparent privacy protocols is essential to ensure the safe adoption of these technologies in care settings for older adults.

### Equity and Access to Care

Equity in access to DHT technologies is another significant concern, especially in the context of older adult care. Although DHTs hold promise for personalized care, disparities in access to technology may exacerbate existing health inequities [16].

#### Technological Barriers Affect Older Care

Older adults, particularly those from disadvantaged socioeconomic backgrounds, may have limited access to the required digital infrastructure or struggle with digital literacy. Furthermore, some older individuals may face technological barriers, such as a lack of internet access or difficulty operating digital devices, which limit their ability to benefit from DHT-based care solutions.

#### Disparities in Health Outcomes

Research studies by Bahrami et al [7,18] highlighted how inequitable access to tailored drug therapies and health interventions could worsen disparities in health outcomes if these technologies were not broadly accessible or affordable. Barbiero et al [11] similarly argued that without equitable distribution and access to DHT-based care models, exacerbating the social determinants of health and creating new forms of inequality in health care delivery were at risk.

To achieve true personalization of care, DHTs must be deployed with an emphasis on overcoming these access barriers, ensuring that all older adults benefit equally from these advancements.

### Effectiveness of DHT Implementation in Relation to Location, Timing, and Context

The location and timing of DHT implementation significantly influence its effectiveness in care settings for older adults. Liu et al [26] and Bruynseels [12] showed that the efficacy of DHTs could vary considerably across different care environments, including home care, nursing homes, and hospital settings.

#### Limited Resources Cause Challenges in DHT Implementation

The technological infrastructure of these settings played a crucial role in determining the feasibility and success of DHT integration. For example, in-home care environments, where resources might be limited, implementing DHTs could be more challenging compared to well-equipped health care facilities [29].

#### Rapid Deployment Leads to Insufficient Validation of DHT Implementation

Furthermore, the timing of DHT deployment can influence its impact. This then emphasized the integration of DHTs during a health crisis (such as the COVID-19 pandemic), accelerated adoption, but also raised challenges related to rapid deployment [21], including insufficient validation and potential overreliance on untested technologies. The timing of DHT use in disease progression was also critical—early-stage interventions, particularly in conditions such as dementia, are most beneficial, as they could significantly improve patient outcomes [34].

Therefore, the contextual environment in which DHTs are deployed must be carefully considered to maximize the benefits of these technologies.

### Ethical Concerns: Autonomy, Consent, and Overdiagnosis

Ethical concerns are central to implementing DHTs, particularly in terms of patient autonomy, informed consent, and the risk of overdiagnosis.

#### Informed Consent Becomes a Complex Ethical Issue

Many older adults, especially those with cognitive impairments, might face challenges in providing informed consent for digital health interventions [30]. Informed consent in these contexts became a complex ethical issue, as health care providers must balance the benefits of personalized care with the need to protect vulnerable patients’ rights to autonomy and decision-making [22,26,28].

#### The Issue of Overdiagnosis Leads to Harmful Side Effects and Unnecessary Treatments

In addition to autonomy and consent, the issue of overdiagnosis was another significant ethical concern. Lin et al pointed out that the predictive capabilities of DHTs might lead to overdiagnosis, particularly in screening programs, resulting in unnecessary treatments and interventions. This concern was especially relevant for older populations [22], where the risks associated with overdiagnosis—such as the potential for harmful side effects or unnecessary treatments—are more pronounced due to comorbidities and frailty [26].

While DHTs offer the potential for early detection, they must be used judiciously to avoid these unintended consequences and ensure that interventions are genuinely beneficial rather than causing harm.

### Impact on Care Workflow and Provider Workload

Introducing DHTs into care settings for older adults may significantly alter the care workflow, with both positive and negative implications.

#### The Need for Enhancing Care Efficiency

Studies by Bahrami et al [7] and Bui [21] highlighted that DHTs could enhance care efficiency by automating specific tasks [21], such as continuous health monitoring, data collection, and decision-making support [7]. These improvements could reduce the burden on health care providers and streamline care coordination. However, integrating these technologies also required adaptation to new systems and workflows, which might lead to initial disruptions and increased workload.

#### The Need for an Increase in Training Requirements for Existing Practices

Furthermore, as health care providers become familiar with DHT technologies, there may be an increase in training requirements and adjustments to existing practices, which could potentially lead to a higher workload in the short term. While the long-term benefits of DHTs, such as reduced manual tasks and improved diagnostic accuracy, are clear, the transition period must be carefully managed to avoid burnout or resistance among health care staff.

Therefore, successfully integrating DHTs into care settings for older adults requires thoughtful planning to balance the expected workflow improvements with the challenges of training and adapting health care staff to new technologies.

### Strategies to Mitigate DHT Challenges

Several studies propose strategies to mitigate these ethical challenges that prioritize inclusivity, transparency, and patient autonomy (see Multimedia Appendix 1). Governance frameworks were aimed at ensuring the data had openness and privacy [12]. On the other hand, Sahal et al [32] and Zhou et al [33] advocated for integrating blockchain technologies and decentralized models to enhance data security and user control. In addition, Vidal et al [28] and Wickramasinghe et al [31] highlighted the importance of multidisciplinary collaboration and integrated care models, such as integrated living models, which promoted coordinated care and supported the ethical implementation of DHTs in practice [28,31].

Collectively, these studies illustrated a shared understanding of both the opportunities and ethical responsibilities associated with the deployment of DHTs in older adult care settings. There has been a consistent call for robust ethical oversight, patient-centered governance, and policies that ensure equitable access to these technologies [12,21,23].

### Further Suggestions

Several studies offer suggestions for improving DHT integration into health care. Most studies highlighted ethical and quality of care challenges in care settings for older adults and recommend developing clear guidelines for using DHT (see Multimedia Appendix 1). For instance, Venkatesh et al [9] suggested considering computation, implementation, and regulation challenges to ensure the proper functioning of health digital twins for precision medicine. These suggestions highlighted the importance of addressing technical and ethical hurdles for DHTs to integrate into health and care systems fully.

## Discussion

### Principal Findings

The implementation of DHTs in health and social care for older adults presents both significant opportunities and inherent risks. In this scoping review, 20 studies were included from a total of 856 identified through a systematic literature search. This review aims to gain insights into the best available evidence on the implementation of DHTs for older adults in health and social care. This discussion critically examines five key challenges that emerge from the literature. These challenges include (1) data security and privacy concerns; (2) equity and accessibility of health care; (3) effectiveness in relation to context, timing, and location; (4) ethical implications regarding autonomy, consent, and overdiagnosis; and (5) the impact of DHTs on health care workflows and provider workload. Overcoming such issues will ensure that DHTs contribute to the quality of care of older individuals and do not take away from it.

The increasing reliance on DHTs requires robust data protection, particularly for vulnerable populations, such as older individuals. DHTs are based on large multimodal datasets, including biometric data, health records, and real-time environmental sensing, which raise significant ethical and privacy concerns [26,30]. Several studies highlight the risks associated with unauthorized data access and potential misuse, particularly for older individuals who may lack familiarity with digital systems and, consequently, are more vulnerable to exploitation [5,12]. Although DHTs provide substantial benefits, such as continuous health monitoring [40] and predictive health care analytics [7], they also introduce risks, including data breaches and unwarranted surveillance [19]. The establishment of ethical guidelines that mandate transparency, accountability, and patient consent is essential in curbing these threats [20]. Blockchain technology, as proposed by Sahal et al [32], could potentially provide a safe manner of data management; nonetheless, empirical investigations are required to ascertain its usefulness in real-life health care settings.

While DHTs offer potential advancements in personalized health care, concerns remain regarding equitable access. The digital divide, particularly amongst older cohorts within lower socioeconomic segments, stands to exacerbate health care disparities [21]. Furthermore, digital twin-controlled drug therapies and individualized health interventions may inadvertently become privileged health care solutions, accessible predominantly to wealthy populations, thereby further aggravating existing disparities [11]. Equity must take precedence while deploying DHTs, with policy measures to curb accessibility barriers through government policies, ring-fenced finances, and digital literacy programs. Otherwise, integrating DHTs will likely widen health and care inequalities rather than bridge them.

The performance of any digital health technology dramatically depends on the environment in which they are implemented [41,42]. Studies show that DHTs perform optimally in well-structured health care settings, such as hospitals and specialist clinics, with strong technological infrastructures that support real-time patient location and accurate data integration [26]. The usefulness of such technologies in home-care environments where technology and care capabilities are limited has not been adequately established [27]. On the other hand, the timing of DHT implementation is also a critical factor influencing its success. The rapid adoption of DHTs during the COVID-19 pandemic raised concerns regarding premature reliance on untested digital health solutions [20]. Similarly, DHT interventions conducted early in the course of dementia care are more beneficial than interventions done in later stages [19], and improve pain management and reduce patient outcome variability with the effect of physiological features on the achieved pain relief [18]. Therefore, strategies for implementing DHT must be customized according to individual health status, disease stages, and diverse care settings to optimize their impact.

The application of DHTs generates complex ethical questions, primarily about patient autonomy and informed consent. Older people with impaired cognition may be unable to fully grasp the implications of consenting to continuous digital health monitoring [29]. In addition, coercion, the compulsion that persons may feel in adopting DHTs by virtue of health care policy or social culture, is of interest to voluntary involvement [21]. The second ethical concern is overdiagnosis [43,44]. DHT predictive algorithms can lead to an overestimation of disease risk and, consequently, unnecessary medical procedures that increase patient anxiety and place additional pressures on health care systems (Lin et al [22]). While early diagnosis is one of the significant advantages of DHTs, careful calibration is necessary to distinguish between necessary interventions and overmedicalization. Ethical guidelines governing the use of DHT must prioritize informed patient choice over algorithmic assumptions, so that clinical decisions are made appropriately based on clinical need [28,45].

The presence of DHTs in geriatric care settings has conflicting effects on health care workers. On the one hand, DHTs can streamline care processes, reducing administrative workload through automated data collection and real-time patient monitoring, thereby enhancing clinical decision-making [7,20]. On the other hand, the transition to DHT-based care models necessitates significant changes to clinical workflows, potentially increasing short-term workload and requiring extensive training [31]. This necessitates that health care professionals be adequately trained to interpret and apply DHT-generated insights to patient care plans. Research by Alves et al [5] suggests that virtual reality (VR)-based training simulations facilitate the integration of digital twin technology before full-scale implementation. However, unless carefully managed, there is a risk of overreliance on automated decision-support systems, potentially undermining the clinical judgment of health care professionals [23]. Consequently, the integration of DHTs must be approached with caution, ensuring a balance between technological efficiency and the human expertise essential to high-quality patient care.

### Strengths and Limitations of the Study

The study provides a strong ethical analysis, but it would benefit from more involvement of health care professionals and older adults, which could have enriched the findings with practical insights. The study highlights the importance of balancing technological innovation with ethical considerations to ensure DHTs enhance, rather than compromise, the quality of care for older adults. One of the key advantages is that this provides valuable insight into the benefits. A key strength is a systematic approach; the guidelines by JBI’s methodology for scoping reviews ensure methodological rigor. This study is comprehensive, as it consolidates evidence from various studies on the safety, equity, practicality, ethics, and impact on care workflow of these digital clinical points of care. This illustrates the potential of DHTs in early disease detection and dementia, particularly in highlighting their potential contribution to preventive health care [19]. However, concerns over overdiagnosed patients and unintended consequences, including heightened anxiety and unnecessary procedures, highlight the lack of empirical validation [22].

A significant limitation is the use of secondary data drawn mostly from the published literature, which may exhibit bias or an evidence gap, particularly in the context of mask implementation for DHTs in practice. Another drawback of the study is that the latest search was conducted in October 2023; no further search could have been performed due to lack of resources, including funding and the research team’s time commitment. For the same reason, that is, the lack of resources, we also did not follow the JBI Methodology strictly, but instead used it as a guide. Although the search identified 786 studies, some may have been missed. Due to the strict adherence to quality assurance in the gray literature search, including the credibility, objectivity, and accuracy of non–peer-reviewed sources, no unpublished articles could have been included in this review, except the study by Bui [21] (thesis) in 2023. Another limitation is that only studies published in English were included. However, the findings of 20 included studies relate to a range of 14 countries worldwide, with most studies emanating from the United Kingdom and Europe.

### Ethical Frameworks and Policy Development

The following key considerations should guide future practice and policy:

- The implications of this study emphasize the necessity for solid ethical guidelines and policy frameworks to mitigate the potential risks associated with DHT application in older adult care and to ensure that the patient remains the ultimate decision-maker [30,46,47].
- Patients’ autonomy and data privacy should be protected while fair access to technology that has the potential to enhance patient care is maintained [20,21].

### Health Care System: Adaptation

To support the effective integration of DHTs into care for older adults, several key considerations for system adaptation can be identified:

- This study clearly highlights the pressing need for health and social care providers and institutions to integrate DHTs thoughtfully [48].
- It is imperative to strike a balance between the benefits offered by these technologies, particularly their predictive and monitoring capabilities. Concerns may then be related to overdiagnosis, increased workloads, and the potential for disparities in care delivery [7,22].
- Limitations associated with relying solely on secondary data could indicate that future research should prioritize empirical validation through direct engagement with health care professionals, caregivers, and older adults [49,50].
- Engagement is essential for refining the practical applications of DHTs and effectively assessing their real-world impact [9,19,29].

### Future Research and Stakeholder Engagement

The following key directions are proposed to guide future research and strengthen stakeholder engagement:

- Given the limitations of relying on secondary data, future studies should prioritize empirical validation through co-design with stakeholders, such as health care professionals, caregivers, and older adults.
- All to refine the practical applications of DHTs and assess their real-world impact [19,29].
- To address stakeholder engagement and practical relevance, the findings can be effectively translated into practice through the development of guidelines and frameworks.
- Further research can examine other dimensions of the quality of care, such as access, timeliness, acceptability, and appropriateness.

### Conclusion

This scoping review was conducted to assess the available evidence on implementing DHTs for the care of older adults, highlighting both significant opportunities and inherent risks. A systematic search of the literature yielded 20 studies from an initial 856 records. The studies revealed several critical characteristics regarding the implementation and ethical considerations of DHT technologies, particularly in terms of safety, equity, timing, location, participant characteristics, and their impact on workflow. A total of 5 key challenges were identified through the integrated synthesis, which is crucial for understanding the potential and limitations of DHTs in enhancing care for older adults. The challenges included (1) data security and privacy concerns; (2) equity and accessibility of health care; (3) effectiveness concerning context, timing, and location; (4) ethical implications regarding autonomy, consent, and overdiagnosis; and (5) the impact of DHTs on health care workflows and provider workload. The implications of these challenges underscore the need for more practical ethical guidelines and policy frameworks to mitigate the potential risks associated with DHT application in older care, ensuring that patients remain the ultimate decision-makers. Further research should be conducted to examine other dimensions of the quality of care, such as timeliness, acceptability, and appropriateness.

## Supplementary material

10.2196/73925 Multimedia Appendix 1 Key characteristics of the included studies.

10.2196/73925 Multimedia Appendix 2 Additional tables on the search strategy on databases and the inclusion and exclusion criteria.

10.2196/73925 Checklist 1 PRISMA-ScR checklist.

## Abbreviations

DHT: digital health twin
JBI: Joanna Briggs Institute
PCC: population, concept, and context
PDT: personal digital twin
PRISMA: Preferred Reporting Items for Systematic Reviews and Meta-Analyses
PRISMA-ScR: Preferred Reporting Items for Systematic Reviews and Meta-Analyses Extension for Scoping Reviews
VR: virtual reality
