# Supplementary material for: Ethical and Quality of Care–Related Challenges of Digital Health Twins in Care Settings for Older Adults: Scoping Review
Source: JMIR Aging. 2025 Oct 28;8:e73925. doi: 10.2196/73925 (PMC12560958; doi:10.2196/73925)
Supplement: Multimedia Appendix 1 [file aging-v8-e73925-s001.docx]

**Table S1: Key characteristics of included studies**

| **No.** | **Authors & Year, Country** | **Aim** | **QL/QT** | **Population/**  **participant: age and gender** | **Setting/Sample size** | **Outcomes measured/Phenomenon of interest** | **Claim/Findings** | **Strategies** | **Further suggestions** |
| --- | --- | --- | --- | --- | --- | --- | --- | --- | --- |
| 1 | Bruynseels et al., 2018  Netherlands | -Exploring the potential of DHT in healthcare settings. | QT |  |  | -Understanding how DHT can improve personalised healthcare  -Conceptual and ethical aspects of future healthcare and human enhancement  -Ethical and societal implications of DHT | -DHT in healthcare raised ethical, legal, and social issues for therapy and enhancement.  -It presented equality issues that might lead to discrimination based on differences in compiled information.  -PDT highlighted the importance of governance for the use of personal data. | Governance for data transparency and privacy |  |
| 2 | Liu et al., 2019  China | -Developing a framework for using cloud digital twin in healthcare settings. | QT |  |  | -Monitoring, diagnosing, and predicting aspects of the health of individuals | -It provided the framework and application methods of DHT in the cloud.  DTH was proposed to address the challenges of real-time supervision and the accuracy of crisis warnings for seniors in healthcare settings.  -The reference framework of CloudDTH was given, and a DTH model was constructed | Cloud-based framework to improve care delivery | -Data integration and fusion; building and managing the DTH models  -Model accuracy verification and model evaluation  -Collaboration and interaction between machines and services  -Accessing different medical devices and sensors using various protocols to the CloudDTH platform |
| 3 | Chakshu et al., 2021  United Kingdom | -Proposing a methodology to detect the severity of carotid stenosis from a video of a human face with the help of a coupled blood flow and head vibration model, which supports non-invasive | QT | Healthy individuals: 20-30 years; virtual patients: 40-80 years |  | -Non-invasive detection of carotid stenosis | -A model that combined computer vision and computational mechanics could detect severe carotid stenosis without requiring invasive procedures.  -The proposed method was feasible but had the potential for significant improvements. The non-invasive procedure was simple and effective. | Semi-active digital twin model for health monitoring | -Emphasis on enhancing the model  -Validating through broader datasets  -Ethical and procedural alignment for practical clinical application  -Move towards an active human digital twin  -Enable continuous monitoring of carotid stenosis/stroke potential |
| 4 | Calderita et al., 2020  Spain | -Verifying the viability of a new cyber-physical system (CPS) that, besides perceiving, can act in the environment and interact with people. | QT |  | Caregiving centres | -Effectiveness of the Cyber-Physical System CPS-AAL in caregiving centres.  -Validation    -Navigation  -Integration of SARs in a smart environment | -The robot presented notable advantages in social navigation behaviour, avoiding situations that were not socially accepted. | CPS-AAL systems for quality-of-life enhancement. | -Extend the use case to cover other essential tasks in a caregiving centre  -Extend CORTEX, and by extension, the digital twin model, with more modelling power and predictive capabilities  -The maintenance of self and world representation in the working memory can be augmented with a temporal dimension in the future and the past  -Inclusion of specialised simulators. |
| 5 | Vidal et al., 2020  France | -Enabling a predictive and preventive approach to managing health and frailty across various living environments and age groups through strategically collecting and analysing diverse data types, utilising a multidisciplinary approach, and considering ethical and societal priorities in healthcare delivery. | QT | 60+ | community, 8 | -Early detection of pre-frailty and frailty  -Appropriate algorithms, faint physiological signals  -Preventive treatment | -It allowed older adults to receive preventive treatment at the correct institutions and by the appropriate professionals as early as possible, which would prevent loss of autonomy | Multidisciplinary collaboration, extending research across all age groups, and enhancing caregiver coordination through ILM | -Extending Research to Various Age Groups as frailty has no age  -Caregiver coordination: ILM to facilitate coordinated actions by caregivers. Develop and refine database (digital twin) for ILM  -Multidisciplinary collaboration among healthcare professionals, engineers, neurophysiologists, and mathematicians is required to develop preventive health policies  -Work on the development and improvement of non-invasive sensors to accurately capture physiological data |
| 6 | Barbiero et al., 2021  United Kingdom | -Shifting from a wait-and-react, curative discipline to a preventative, interdisciplinary science.  -Aiming to provide patients with personalised, systemic, and precise treatment plans.  -Proposing a “digital twin” of patients modelling the human body as a whole and providing a panoramic view of individuals’ conditions | QT |  | 838 | -A general framework that provides a panoramic view of individuals’ conditions | -It developed an easy-to-understand digital model that comprehensively overviewed a patient’s condition. It also demonstrated how physiological models and molecular information could be represented and integrated using GNNs and generative adversarial networks.  -This provided modularity and scalability across layers of biomedical data. It also adapted to various modelling approaches and generated integrated predictions that translated into patients’ trajectories. | Framework as a strategy for preventative medicine |  |
| 7 | Jovanovic et al., 2021  Serbia | -Presenting a novel model for vaccination strategy simulation.  While the model was developed with COVID-19 vaccination as an immediate use case, it could be helpful in several additional scenarios. The non-exhaustive list includes later revaccination strategies and studies of information propagation of other public health-relevant information products. | QT | healthcare workers, adults over 65years | Households, pubs, schools, hospital -2730 | -Disease prevalence  -Decision on vaccination | -Social parameters within stochastic models significantly influenced infection spread predictions.  -This enhanced modelling accuracy through more social groups and increased graph complexity.  -Vaccination prioritisation for younger adults correlated with reduced COVID-19 cases and mortality.  -This increased herd immunity is associated with lower infection and death rates during the COVID-19 outbreak. -Strict public health measures contributed to decreased infection rates and further mortality reduction.  -A novel two-layer graph framework improved vaccination scenario simulations and policy decision-making.  -The study's findings supported targeted vaccination strategies and effective public health interventions to manage COVID-19. | Vaccination strategy simulation model | -Increase in graph complexity, including population information and applying the developed model to other cities in RS, as well as to the whole population of RS, and in the final stage to other countries if their population data are available |
| 8 | Kobayashi et al., 2021  Japan | -Proposing a system that detects signs of dementia without relying on interviews by doctors. | QL |  | Hospital, home | -Cognitive function disorder  -Life function disorder | -The system could digitally transform information required for detecting “cognitive function disorder” and “life function disorder” from daily life, and configure the subjects’ digital twin with high accuracy | Early detection system for dementia care. | -Improving the system's accuracy by incorporating additional factors to influence the onset of dementia and testing the system in more extensive and diverse populations  -Ethical and legal implications of using such a system, including privacy, consent, and data security |
| 9 | Khan et al., 2022  United Kingdom | -Utilising microwave-sensing technology in a static care-home model created in the microwave-modelling tool of CST Studio Suite to collect data unobtrusively.  -Providing insights into sensor placement, performance, and data collection capability in a healthcare environment. | QT |  | Static care home | -Performance  -Collect DT data sets in a static care-home model | -An unobtrusive microwave sensor was analysed for its performance and its ability to collect DT data sets in a static care-home model.  -The position of the sensors was essential to sense the presence of and collect vital patient data.  -Correct data collection was essential for patient diagnosis and the creation of accurate DT models. | Microwave sensing for non-intrusive data collection | -The future work will utilise the realistic patient DHT data collected through unobtrusive microwave sensors with pre-processing and AI—ML algorithms to create a DT model for the autonomous detection of patients’ vital signs, tumours, diseases, and gait. |
| 10 | Bahrami et al., 2022  Switzerland | -Developing a physics-based digital twin for a virtual patient that considers the drug diffusion from the transdermal patch through the skin layers to reach the blood circulation. | QT | 20 – 80-year-old virtual patients |  | -Real-time feedback  -Tailored therapy | -A physics-based digital twin for fentanyl transdermal therapy could anticipate the outcome of conventional therapy on patients of different ages and propose an alternative treatment based on patient feedback.  -The proposed twin-assisted therapy can significantly reduce pain scores and increase the time without pain compared to conventional treatment.  The study also found that incorporating patient feedback into the digital twin could better tailor therapy to real-time patient needs and help avoid adverse treatment effects.  -Patient-specific digital twins could provide more accurate and safer therapies and can be used to propose therapies based on patient needs.  -It provided a roadmap for implementing such twin-assisted treatment clinics and highlighted the added value of physics-based digital twins in personalised medicine. | Physics-based digital twin for personalised medicine. |  |
| 11 | Wickramasinghe et al., 2022  Australia | -Highlighting the potential benefits of incorporating digital twin technology to support better dementia care. | QT | Clinicians, careers, dementia patients |  | -Early intervention  -Precision  -Personalisation | -It found two potential use cases for the framework for clinicians and caregivers | Clinical decision support model for dementia care. |  |
| 12 | Sahal et al., 2022  Ireland | -Introducing the concept of a personal digital twin (PDT) as an enhanced version of the digital twin (DT) with actionable insight capabilities. Explore its potential applications in the healthcare industry.  -Empowering competent, personalised healthcare using PDTs by integrating existing advanced technologies. | QT |  |  | -Integration of DT, AI, blockchain technologies,  -Personalised healthcare services | -Introduction of the PDT concept  -Reference framework as a step towards the smart, personalised healthcare industry. -Described the selected personalised healthcare use cases, | Reference framework for personalised healthcare. |  |
| 13 | Zhou et al., 2022  China | -Analysing the application scenarios and paradigms of the metaverse in medicine in mental health. | QT |  | Four scenarios | -The application scenarios of the metaverse in medicine | -It predicted that the metaverse would break the rules of geriatric diagnosis and treatment habits. The proportion of self-service and remote diagnosis and treatment would increase. Hospitals' management role would weaken, and they would become distributed nodes, losing their central position.  -As more data were added to the metaverse in medicine, this new paradigm became an important preventive tool for diagnosing and treating cognitive decline and AD. | Metaverse as a strategy for non-pharmacological interventions |  |
| 14 | Alves et al., 2022  Canada | -Developing a VR simulator for robotics navigation and fall detection with a digital twin as a solution to test the virtual robot without having access to the real physical location or real people.  -Addressing the challenges posed by the current COVID-19 pandemic has made in-person data collection difficult, especially in long-term care facilities. | QT |  |  | -Virtual reality versus real-world testing | This improved the simulator's performance without sacrificing accuracy. It also saved time and costs associated with real-world testing. | VR simulation for training without patient data | -Adapting the simulator to produce synthetic images and corresponding annotations to create a data set for improving YOLOv4 detection of fallen people  -Use other environments and changes to the scene during runtime to test the robot's performance and detection capabilities, response  -Testing of various human-robot interactions in addition to AI-driven virtual avatars to automate the mobile robot virtual training process |
| 15 | Bahrami, et. al., 2023  Switzerland | -Determining the effect of physiological features on the achieved pain relief. Therefore, a virtual patient set was developed using Markov chain Monte Carlo (MCMC) based on actual patient data. | QT | Ten men, ten women. 40 to 68 years | 3000 virtual cancer patients, sample data 20 cancer patients | -Turnaround time | The digital twin successfully anticipated patient needs and adapted therapy accordingly, enabling tailored treatment strategies and monitoring to improve pain management and reduce patient outcome variability. | Digital twin control of therapy for pain management |  |
| 16 | Bui, N. P. K. 2023  Netherlands | -Providing healthcare executives with insights into seniors’ perceptions, attitudes, and concerns towards DTH. | QT | 0 >80 | 131 | -The seniors’ perception and attitudes towards DHT | -Performance expectancy was the most influential factor in both DTH usage levels, followed by social influence and facilitating conditions in DTH usage level 1.  -For DTH usage level 2, performance expectancy was the most significant factor, while price value was an inhibitor.  -A significant moderating effect of nationality on performance expectancy in DTH usage level 1 and social influence in DTH usage level 2 was found.  -The study concluded that the factors influencing the use intention of DTH were divergent for each level and recommended studying other factors to improve the predictive power of behavioural intention for each DTH usage level. | Behavioural intention understanding for DHT adoption | -Improving the reliability of the research by collecting more prominent and representative samples, using cross-country observations, and retesting to observe whether the same outcomes were produced.  -Extending the UTAUT2 model with medical service satisfaction, physical comfort, product design, human interaction value, self-actualisation, self-efficacy, technology anxiety, resistance to change, and data privacy  -The perspective of healthcare professionals need also be considered as investigating the factors. Studies on the continuous usage of older people and healthcare professionals are highly recommended to reap the benefits of DTH fully. |
| 17 | Lin et al., 2023  Taiwan | -Estimating the proportion of overdiagnosis in population-based service screening programs for CRC with the faecal immunochemical test (FIT)  -Building up the overdiagnosis-embedded disease natural process governed by transition parameters and the calibrated sensitivity while accounting for competing mortality | QT | 50 to 69 | 5,417,699 | -Healthcare effectiveness and ethical considerations. | -The FIT test was less likely to lead to unnecessary colonoscopies and treatments when offered in a population-based CRC service screening program. | Digital twin approach to mitigate overdiagnosis |  |
| 18 | Zhao et al., 2023  China | Investigating the views and expectations of older adults towards intelligent technology that caters to their real needs, particularly those who live independently in their own homes.  -Comparing the effectiveness and perceptions of using the RCSSH system versus smartphone video calls to assist seniors in learning new life skills. | Mixed study | Five men and seven women aged between 57 and 73 with no visual or auditory impairment | 12 | -Older adults' perceptions and expectations towards intelligent technology, | -The remote collaboration system for smart home (RCSSH) was more efficient than traditional cell phone calls for older adults in completing various tasks.  -System: The system did not enhance intimacy due to its first-person view lacking facial displays.  -Older adults showed a positive inclination towards new technologies, but acceptance varied individually, and some technical flaws in the system hindered the optimal user experience | Digital twin remote collaboration enhancement | -Recruiting a diverse set of aged participants, expanding the study to cover a broader range of smart technologies, examining digital twin remote collaboration, using the technology for a prolonged period in a natural setting, and addressing technical flaws to enhance the user experience |
| 19 | Thamotharan et al., 2023  India | -Introducing and demonstrating the efficacy of an HDT framework for managing Elderly Type-2 Diabetes (E-T2D). | QT | 36 to 80 years old patients with type 2 diabetes | 15 | -HDT efficacy.  -Personalised recommendations | -HDT-based recommendations significantly improved in time-in-range (TIR) with fewer hyper and hypo conditions. | HDT framework for individualised diabetes treatment | -Extending the clinical trials for more co-morbid patients and studying it for many populations is the future course of this study |
| 20 | Cai et al., 2023  United States | -Proposing a novel DL framework, named band-dependent learning (BDL), to automatically assess the ADRD risk by identifying the salient features along frequency and time ranges in radar spectrograms. | QT | Animation | 8000 | -Risk evaluation  -Preclinical tool | -This supported the effectiveness and feasibility of the system in terms of the quality of the ADRD risk assessment | STRIDE with MDR and DL for non-invasive risk evaluation | -Improving the model and simulation environment |

AD - Alzheimer’s Disease; ADRD - Alzheimer’s Disease and Related Dementias (referring to conditions affecting memory and cognitive function); ADLs - Activities of Daily Living; AI - Artificial Intelligence
BDL - Band-Dependent Learning (the novel framework being proposed); BPM - Business Process Management; CFR - Code of Federal Regulations; CPOE - Computerized Physician Order Entry; CRC - Colorectal Cancer
CST - Computer Simulation Technology; CST Studio Suite - Computer Simulation Technology Studio Suite; COVID-19 - Coronavirus Disease 2019; CPS - Cyber-Physical System

CPS-AAL - Cyber-Physical System for Ambient Assisted Living; CORTEX - Cognitive Operations Research and Technology Exchange; DL - Deep Learning; DHT - Digital Health Twin; DTH - Digital Twin Health
DT - Digital Twin; EHR - Electronic Health Record; EMR - Electronic Medical Record; E-T2D - Elderly Type-2 Diabetes; FDA - Food and Drug Administration; FIT - Faecal Immunochemical Test; GNNs - Graph Neural Networks
HCI - Human-Computer Interaction; HDT - Health Digital Twin; HDT - Human Digital Twin; ILM - Intelligent Layer Model; IoMT - Internet of Medical Things; IoT - Internet of Things; KPI - Key Performance Indicator
ML - Machine Learning; MCMC - Markov Chain Monte Carlo; NDA - New Drug Application; NLP - Natural Language Processing; PDT - Personal Digital Twin; QT – Quantitative; QL – Qualitative

RCSSH - Remote Collaboration System for Smart Homes; R&D - Research and Development; RS - (Specific region or country context needed); SARS-CoV-2 - Severe Acute Respiratory Syndrome Coronavirus 2

SARs - Sensor-Enabled Autonomous Robots; TIR - Time-in-Range; UTAUT2 - Unified Theory of Acceptance and Use of Technology 2; VR - Virtual Reality; YOLOv4 - You Only Look Once version 4

**References**

1. Bruynseels K, Santoni de Sio F, van den Hoven J. Digital twins in health care: ethical implications of an emerging engineering paradigm. Front Genet. 2018;9:31. [doi: 10.3389/fgene.2018.00031] [Medline: 29487613].
2. Liu Y, Zhang L, Yang Y, et al. A novel cloud-based framework for the elderly healthcare services using digital twin. IEEE Access. 2019;7:49088-49101. [doi: 10.1109/ACCESS.2019.2909828].
3. Chakshu NK, Sazonov I, Nithiarasu P. Towards enabling a cardiovascular digital twin for human systemic circulation using inverse analysis. Biomech Model Mechanobiol. Apr 2021;20(2):449-465. [doi: 10.1007/s10237-020-01393-6] [Medline: 33064221].
4. Calderita LV, Vega A, Barroso-Ramírez S, Bustos P, Núñez P. Designing a cyber-physical system for ambient assisted living: a use-case analysis for social robot navigation in caregiving centers. Sensors (Basel). Jul 18, 2020;20(14):4005. [doi: 10.3390/s20144005] [Medline: 32708496].
5. Vidal PP, Vienne-Jumeau A, Moreau A, et al. An opinion paper on the maintenance of robustness: Towards a multimodal and intergenerational approach using digital twins. Aging Med (Milton). Sep 2020;3(3):188-194. [doi: 10.1002/agm2.12115] [Medline: 33103039].
6. Barbiero P, Viñas Torné R, Lió P. Graph representation forecasting of patient’s medical conditions: toward a digital twin. Front Genet. 2021;12:652907. [doi: 10.3389/fgene.2021.652907] [Medline: 34603366].
7. Jovanović R, Davidović M, Lazović I, Jovanović M, Jovašević-Stojanović M. Modelling voluntary general population vaccination strategies during COVID-19 outbreak: influence of disease prevalence. Int J Environ Res Public Health. Jun 8, 2021;18(12):6217. [doi: 10.3390/ijerph18126217] [Medline: 34201285].
8. Kobayashi T, Fukae K, Imai T, Arai K. Dementia sign detection system using digital twin. Presented at: 2021 Ninth International Symposium on Computing and Networking (CANDAR); Nov 23-26, 2021; Matsue, Japan. [doi: 10.1109/CANDAR53791.2021.00025].
9. Khan S, Saied IM, Ratnarajah T, Arslan T. Evaluation of unobtrusive microwave sensors in healthcare 4.0-toward the creation of digital-twin model. Sensors (Basel). Nov 5, 2022;22(21):8519. [doi: 10.3390/s22218519] [Medline: 36366218].
10. Bahrami F, Rossi RM, Defraeye T. Predicting transdermal fentanyl delivery using physics-based simulations for tailored therapy based on the age. Drug Deliv. Dec 2022;29(1):950-969. [doi: 10.1080/10717544.2022.2050846] [Medline: 35319323].
11. Wickramasinghe N, Ulapane N, Andargoli A, et al. Digital twins to enable better precision and personalized dementia care. JAMIA Open. Oct 2022;5(3):ooac072. [doi: 10.1093/jamiaopen/ooac072] [Medline: 35992534].
12. Sahal R, Alsamhi SH, Brown KN. Personal digital twin: a close look into the present and a step towards the future of personalised healthcare industry. Sensors (Basel). Aug 8, 2022;22(15):5918. [doi: 10.3390/s22155918] [Medline: 35957477].
13. Zhou H, Gao JY, Chen Y. The paradigm and future value of the metaverse for the intervention of cognitive decline. Front Public Health. 2022;10:1016680. [doi: 10.3389/fpubh.2022.1016680] [Medline: 36339131].
14. Alves SFR, Uribe-Quevedo A, Chen D, Morris J, Radmard S. Developing a VR simulator for robotics navigation and human robot interactions employing digital twins. Presented at: 2022 IEEE Conference on Virtual Reality and 3D User Interfaces Abstracts and Workshops (VRW); Mar 12-16, 2022; Christchurch, New Zealand. [doi: 10.1109/VRW55335.2022.00036].
15. Bahrami F, Rossi RM, De Nys K, Defraeye T. An individualized digital twin of a patient for transdermal fentanyl therapy for chronic pain management. Drug Deliv Transl Res. Sep 2023;13(9):2272-2285. [doi: 10.1007/s13346-023-01305-y] [Medline: 36897525].
16. Bui K. Factors influence the behavioural intention to use digital twin healthcare by the elderly: a cross-sectional survey. Tilburg University. 2023. URL: https://arno.uvt.nl/show.cgi?fid=161529 [Accessed 2025-10-25].
17. Lin TY, Chiu SYH, Liao LC, Chen SLS, Chiu HM, Chen THH. Assessing overdiagnosis of fecal immunological test screening for colorectal cancer with a digital twin approach. NPJ Digit Med. Feb 10, 2023;6(1):24. [doi: 10.1038/s41746-023-00763-5] [Medline: 36765093].
18. Zhao Y, Mao M, Wang Y. Research on the elderly user assisted experience based on digital twin remote collaboration system. In: Design Studies and Intelligence Engineering. IOS Press; 2023. [doi: 10.3233/FAIA220717].
19. Thamotharan P, Srinivasan S, Kesavadev J, et al. Human digital twin for personalized elderly type 2 diabetes management. J Clin Med. Mar 7, 2023;12(6):2094. [doi: 10.3390/jcm12062094] [Medline: 36983097]
20. Cai F, Patharkar A, Wu T, Lure FYM, Chen H, Chen VC. STRIDE: Systematic radar intelligence analysis for ADRD risk evaluation with gait signature simulation and deep learning. IEEE Sens J. May 15, 2023;23(10):10998-11006. [doi: 10.1109/jsen.2023.3263071] [Medline: 37547101].
