# Supplementary material for: Ethical and Quality of Care–Related Challenges of Digital Health Twins in Care Settings for Older Adults: Scoping Review
Source: JMIR Aging. 2025 Oct 28;8:e73925. doi: 10.2196/73925 (PMC12560958; doi:10.2196/73925)
Supplement: Multimedia Appendix 2 [file aging-v8-e73925-s002.docx]

**Table S2: Search strategy on Databases [8]**

| **Participant, concept, & context scheme** | **#** | **Search string** | **Hits on MEDLINE (July 18)** | **Hits on CINAHL (July 18)** | **Hits on APA PsycInfo (July 18)** | **Hits on Web of Science (July 18)** | **Hits on Scopus (July 19)** | **Hits on WorldCat (July 20)** | **Hits on JBI (July 20)** |
| --- | --- | --- | --- | --- | --- | --- | --- | --- | --- |
| Digital health twin in older care settings | 1 | "digital health twin*" OR "digital twin*" OR "digital phenotype" OR "digital shadow" OR "virtual patient*" OR "personalised health model*" OR "digital patient model" OR "in silico patient*" | 6,404,833 | 639 | 298) | 11,763 | 17,051 | 36,108 | 2 |
|  | 2 | older OR aged OR elderly OR senior* OR elder OR "old person*" OR "older person*" OR "old people" OR "older adult*" OR "older people" OR geriatric* | 2,457 | 1,251,839 | 829,113 | 5,690,683 | 6,817,585 | 31,208,688 | 1,773 |
|  | 3 | # 1 AND #2 | 204 | 46 | 21 | 449 | 327 | 1,682 | 0 |
| Health care setting | 4 | health* OR hospital* OR care* OR caring OR nursing OR treatment OR aid OR management OR therapy | 18,297,577 | 5,152,991 | 2,786,771 | 21,428,220 | 23,457,440 | 5,180 | 2,837 |
| Combined | 5 | #3 AND #4 | 189 | 43 | 19 | 308 | 250 | 1,384 | 0 |
| Filters | 6 | #3 + English, from 2002 onward (exc meeting abstract, editorial material and book chapters. | 197 | 45 | 21 | 428 | 309 | 8 | 0 |
| Filters | 7 | #5 + English, from 2002 onward (exc meeting abstract, editorial material and book chapters. | 184 | 43 | 19 | 300 | 237 | 4 | 0 |

**Table S3: Inclusion and exclusion criteria [8]**

| **Feature** | **Inclusion criteria** | **Exclusion criteria** |
| --- | --- | --- |
| Article type | · Review articles · Conference paper · Grey literature · Early Access | · Meeting abstracts · Editorial materials · Book chapters |
| Language | · English | · All other languages |
| Participants | · Studies involving older individuals associated with DHT, regardless of gender, age, ethnicity, socio-economic status, disorders, or disability. · Studies that include paid or unpaid carers, whether they are family members or friends. · Studies that include care providers involved in older care and DHT, whether they are licensed or unlicensed. | Studies that do not involve older individuals or care providers associated with DHT in older care settings |
| Concept | Studies that evaluate and discuss the process and application of DHT involving caregivers, older individuals, or family/friends/relatives | Studies that do not focus on the application of DHT or are not directly related to older care settings |
| Year | Studies from 2002 through 2023 | Studies from 2001 and earlier |
| Context | Studies that specifically evaluate and discuss the process and application of DHT in settings such as geriatric wards of primary healthcare, hospitals or clinics, nursing homes, care homes, and home care facilities for older individuals | · Studies not in the context of older care ·Studies where DHT is not used. |
